# Transarterial embolization of radicular arteriovenous fistula at the craniocervical junction

**DOI:** 10.1016/j.radcr.2024.01.053

**Published:** 2024-02-13

**Authors:** Songhyon Lee, Masaaki Kubota, Yosuke Tajima, Iori Kojima, Yoshinori Higuchi

**Affiliations:** a Department of Neurological Surgery, Graduate School of Medicine, Chiba University, Chiba, Japan; b Department of Neurosurgery, Narita Red Cross Hospital, Chiba, Japan

**Keywords:** Radicular AVF, CCJ, TAE, Selective angiography

## Abstract

Craniocervical junction arteriovenous fistula (CCJ AVF) is a rare vascular disorder. Direct surgery for CCJ AVF is generally reported to have better outcome compared to endovascular treatment. However, no certain consensus has been obtained so far. We report a case of radicular CCJ AVF treated by transarterial embolization that resulted in a good outcome. A 69-year-old man presented with subarachnoid hemorrhage primarily in the posterior cranial fossa. Based on digital subtraction angiography showed radicular CCJ AVF with varix. Transarterial embolization was performed with n-butyl-2-cyanoacrylate on day 17 after onset and successfully cured. The neurovascular anatomy of CCJ AVF is complicated, but endovascular treatment may be a treatment option with detailed understanding of angioarchitecture and selective endovascular procedure.

## Introduction

Craniocervical junction arteriovenous fistulas (CCJ AVFs) are extremely rare vascular malformations presented with subarachnoid hemorrhage (SAH) or myelopathy [1,2]. Among them, radicular AVF (RAVF) has a shunt point on the spinal nerve root and accounts for approximately 29% of CCJ AVFs [1,3]. Due to their complex neurovascular anatomy, as well as difficult catheter access, endovascular treatment generally requires retreatment or leads to neurological complications. Here, we describe a case of endovascular transarterial embolization of RAVF at CCJ presented with SAH resulted in a favorable outcome.

## Case presentation

**Fig. 1:**
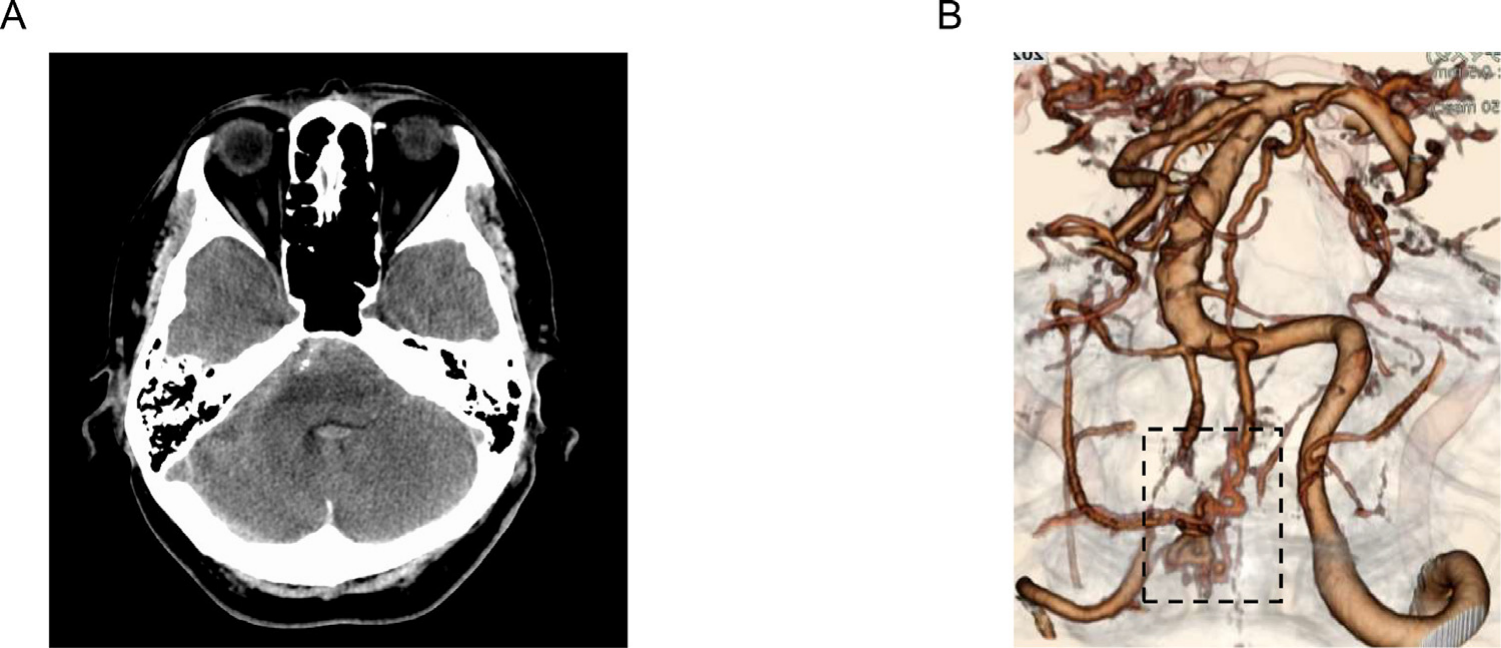
(A) Computed Tomography image shows that the subarachnoid hemorrhage (SAH) is mainly located in the right pontocerebellar cistern. (B) Computed tomography angiography shows abnormal blood vessels around the posterior cranial fossa (area marked by dotted lines).

A 69-year-old man was transported to his previous physician due to sudden onset posterior neck pain. His Glasgow Coma Scale (GCS) score was 15 and he had no neurological deficit. Computed tomography (CT) showed subarachnoid hemorrhage (SAH) in the posterior cranial fossa especially around right pontocerebellar cistern (Fig. 1A). The World Federation of Neurosurgical Society (WFNS) grade was 1, Hunt, and Kosnik grade I, Fisher group was 3. CT angiography revealed tortuous vascular malformation in the posterior cranial fossa (Fig. 1B), and transferred to our hospital on day 2 after onset for further evaluation and treatment.

**Fig. 2:**
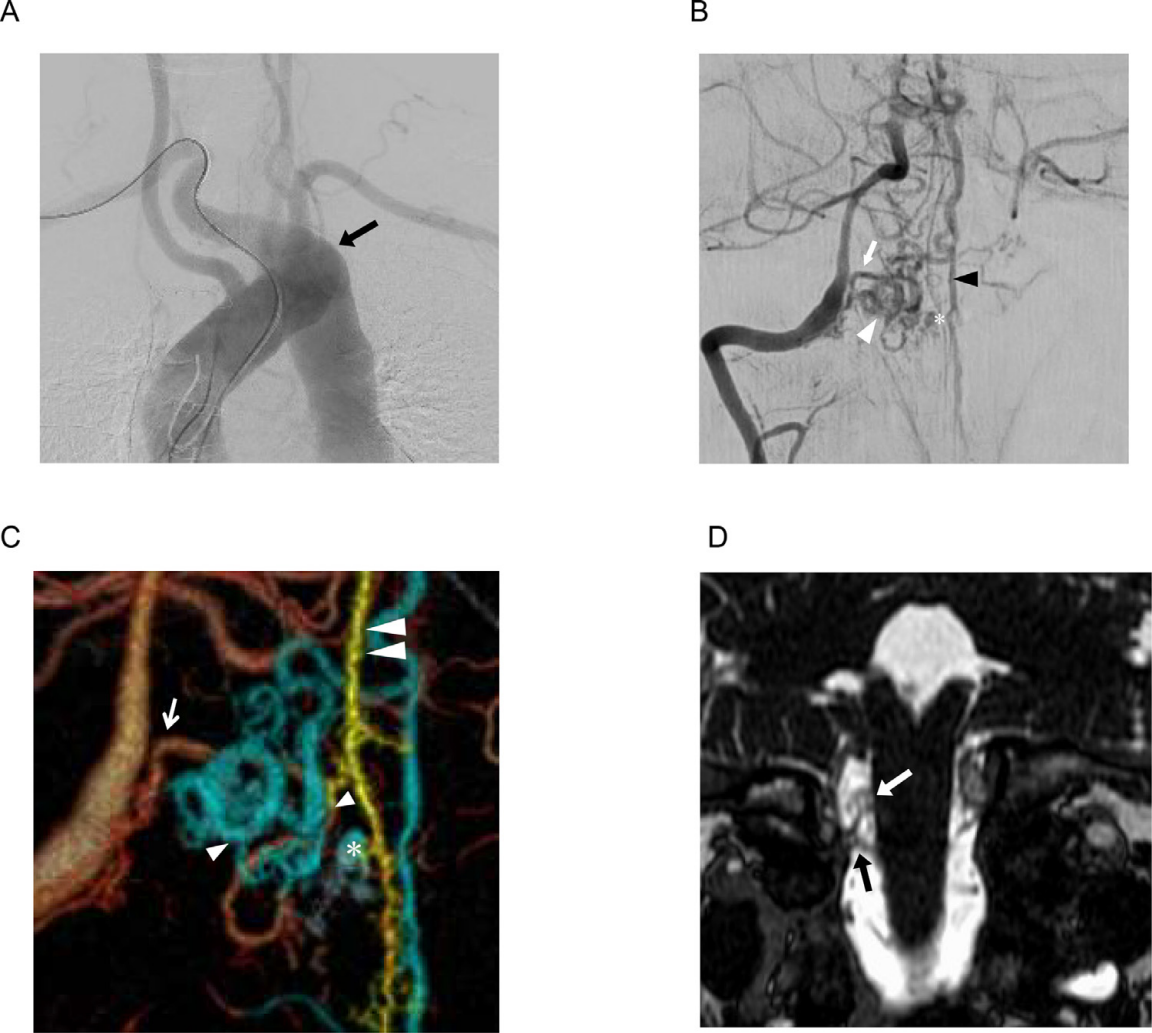
(A) Aortography shows an aberrant right subclavian artery (black arrow) originating from the descending aorta. (B) Right vertebral angiography from anterior-posterior view. The angiography shows a right C1 radicular artery (white arrow), a feeder of the AVF. A shunted point where there is a caliber change is indicated in white arrowhead. A varix is shown in asterisk and anterior spinal vein is shown in black arrowhead. (C) 3D-RA fusion image of right vertebral angiography and left vertebral angiography (white arrow: right C1 radicular artery, double arrowhead: anterior spinal artery, white arrowhead: shunt point, asterisk: varix). (D) Heavy T2 weighted image shows the fistula is recognized on the C1 nerve root (black arrow: C1 nerve root, white arrow: fistula).

We performed digital subtraction angiography (DSA) to evaluate the vascular malformation. Aortography revealed an aberrant right subclavian artery (ARSA), a rare variation of aorta. Right vertebral artery (VA) angiography demonstrated an arteriovenous shunt and a varix. The shunt was fed by the right radicular artery (RA) from C1 and the anterior spinal artery (ASA). Three-dimensional rotational angiography (3D-RA) fusion image of the right VA angiography and the left VA angiography showed that both feeder arteries had a shunt point, and they ascendingly drained through the anterior spinal vein (ASV) (Figs. 2A-C). Heavily T2-weighted magnetic resonance image (MRI) revealed that the fistulous point was situated in the vicinity of the right C1 nerve root (Fig. 2D). Based on these findings, we diagnosed the patient with RAVF at CCJ. We considered the varix that had occurred on one of the drainer veins to be the source of the hemorrhage, and embolization was performed 17 days after onset to prevent rebleeding.

**Fig. 3:**
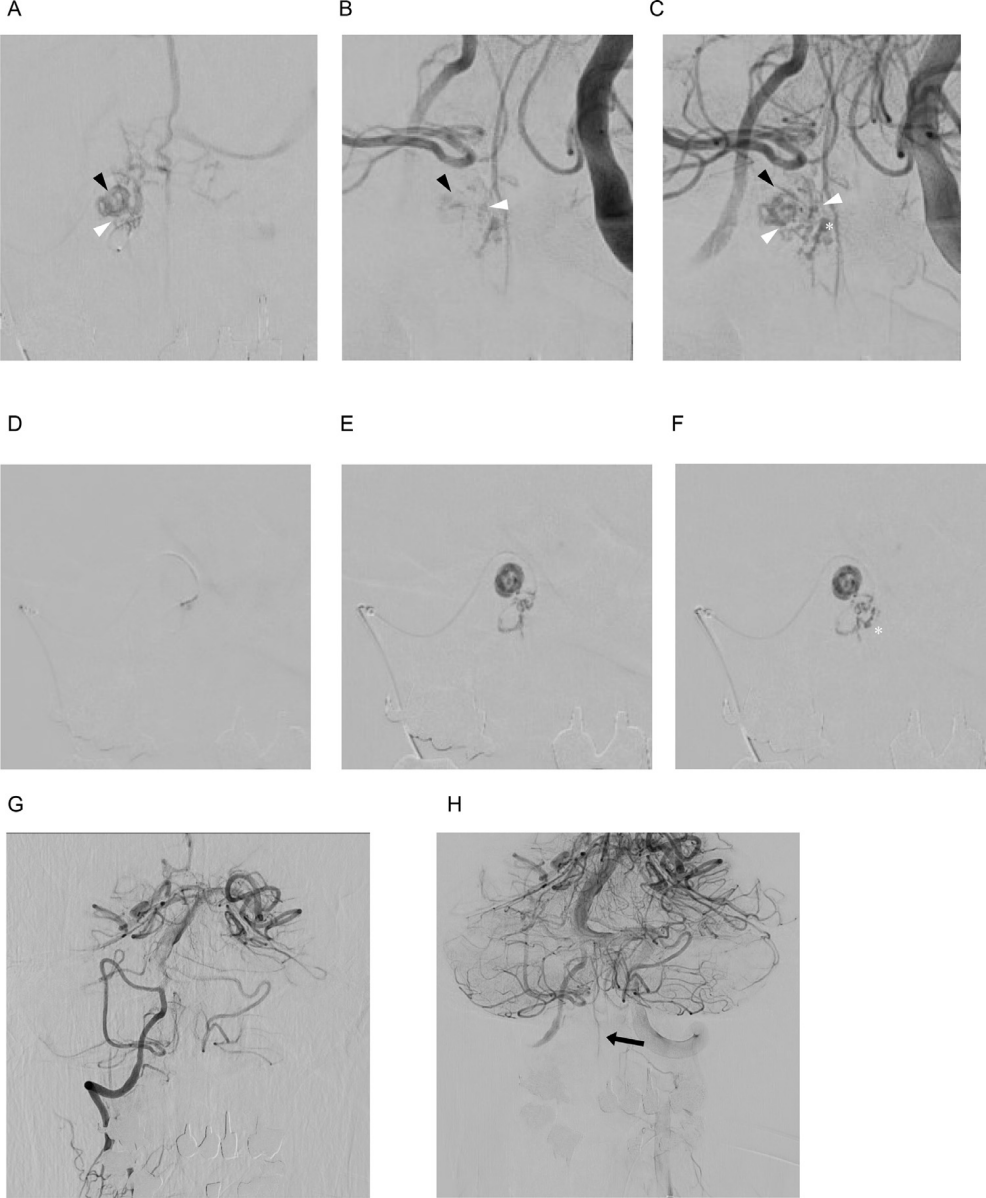
(A) Selective angiography via a microcatheter advanced into a feeding artery originating from the radicular artery (RA) shows the shunt points (white arrowhead: shunt point, black arrowhead: draining vein leading to anterior spinal vein). (B, C) Left vertebral angiography (B: early phase, C: late phase) shows the ASA feeds the arteriovenous fistula (white arrowhead: shunt point, black arrowhead: draining vein, asterisk: varix). (D-F)33% n-butyl-2-cyanoacrylate (NBCA) is injected to the arteriovenous fistula from the right C1 radicular artery (asterisk: varix). (G) Post-procedural right vertebral angiography shows disappearance of the arteriovenous fistula. (H) Postprocedural left vertebral angiography shows normal perfusion of the anterior spinal artery (black arrow).

The procedure was performed under general anesthesia. Due to the ARSA, a 4Fr guiding sheath (FUBUKI Dilator Kit, Asahi Intecc Co Ltd, Aichi, Japan) was delivered to the right VA via the right radial artery approach. Another 4Fr guiding catheter (FUBUKI Dilator Kit) was inserted through the left radial artery. Then, a Scepter XC (MicroVention Inc., Tustin, CA, USA) was placed into the left VA for flow control of ASA by balloon catheter. We used a distal access catheter (Guidepost, Tokai Medical Products, Aichi, Japan), and inserted a micro-guidewire (CHIKAI10, Asahi Intecc Co Ltd, Aichi, Japan) and micro-catheter (De-Frictor Nano, Medico's Hirata, Osaka, Japan) into the right C1 RA. Based on selective angiography of the RA and the left VA angiography, the shunt points were different from each other. Therefore, we determined that flow control of the ASA using a balloon catheter in order to achieve adequate embolization was unnecessary (Figs. 3A-C). We decided to finish the embolization as soon as the drainer vein connected to the varix was visualized.

For the embolization, we selected n-butyl-2-cyanoarcrylate (NBCA, B. Braun Surgical, SA, Rubi, Spain), with a concentration of 33%. The NBCA was injected carefully under 3 fps imaging under a blank road map, and the De-Frictor Nano was removed immediately after confirming that the NBCA reached the drainer vein connected to the varix (Figs. 3D-F). Angiography confirmed that the shunt and the varix had disappeared and that blood flow in the ASA was intact, and the procedure was completed (Figs. 3G and H).

**Fig. 4:**
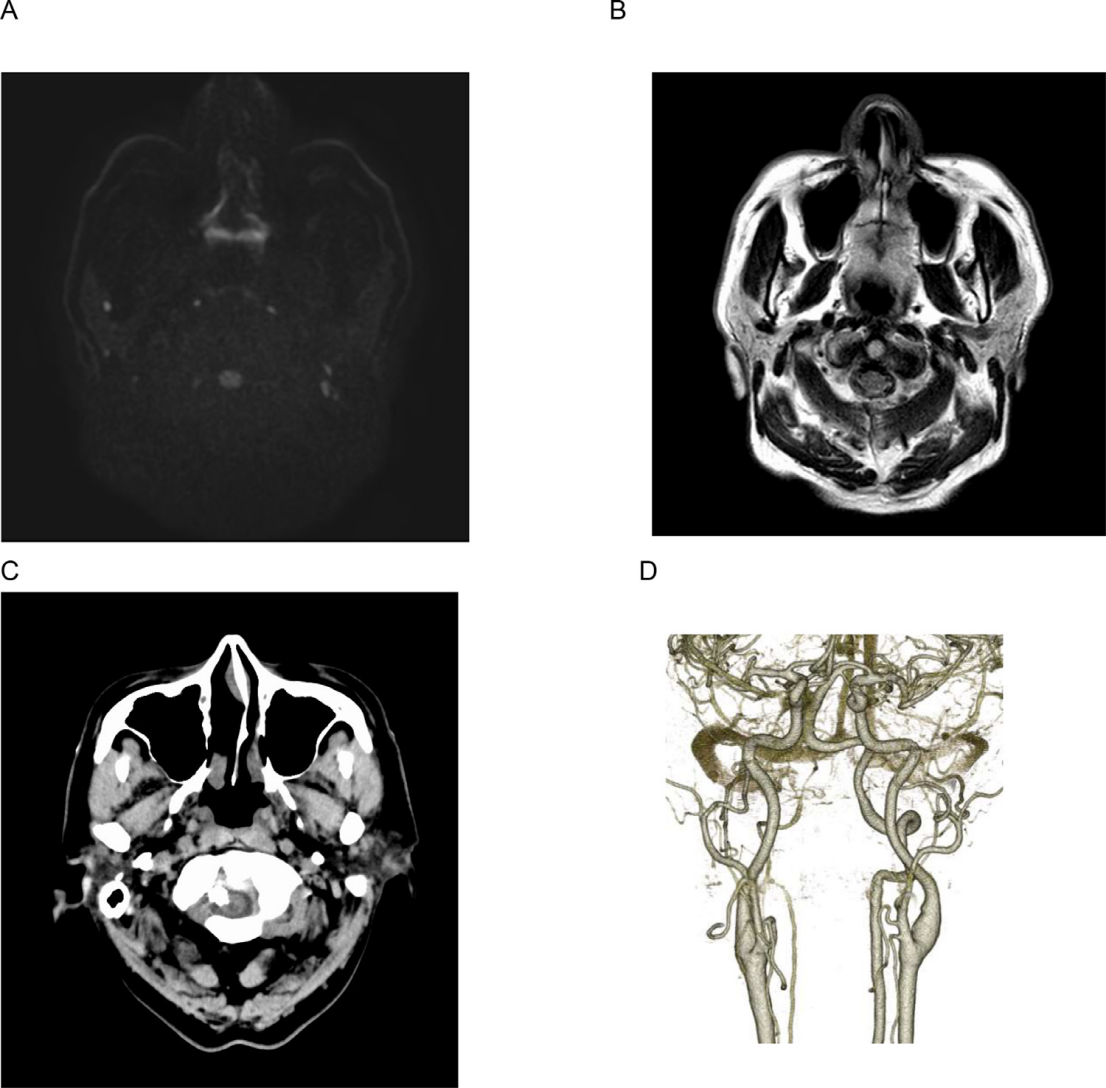
(A) A postprocedural MRI-DWI shows no evidence of spinal infarction. DWI: diffusion weighted image. (B) A postprocedural MRI-FLAIR shows no evidence of high intensity lesions. FLAIR: fluid attenuated inversion recovery. (C) A postprocedural CT image shows no evidence of hemorrhage and the presence of NBCA casts along the spinal nerve root. (D) Postoperative CT angiography image confirmed no evidence of abnormal blood vessels around the posterior cranial fossa.

Post-operative MRI showed no signs of infarction (Figs. 4A and B) and CT showed NBCA cast on the surface of the spinal cord (Fig. 4C). Additionally, CTA revealed disappearance of the vascular lesion. Postoperatively, residual numbness localized to the left tibia, but no other neurological abnormalities were observed (Fig. 4D). The patient was discharged 9 days postintervention with modified Rankin Scale (mRS) score 1. One-year follow-up angiography showed complete obliteration of the fistula.

## Discussion

The CCJ AVF is a rare shunt disease with a frequency of 1%-2% of patients with intracranial or spinal AVF, and RAVF is found in 29% of CCJ AVF [1,3]. SAH, intramedullary hemorrhage, and myelopathy are known as the common clinical presentation [1,2], and drainage patterns play an important role in these presentations. Historically, the presence of ascending venous drainage, varix of drainage vein, RAVF, and arterial feeder of ASA have been suggested significantly high-risk factors for SAH [4], [5], [6], and these were also observed in our case. Also, we recognized ARSA in which the right subclavian artery arose from the descending aorta [7]. This condition is one of the most common aortic arch anomalies found in 0.4%-2% [8]. When ARSA is present, transradial, or transbrachial approach of the right side makes it easier to access to the right vertebral artery [9]. Therefore, we selected transradial approach in the present case.

Principle of the treatment of CCJ AVF is obliteration of the fistula and source of the hemorrhage. Direct surgery and endovascular treatment are optional therapeutic management for CCJ AVF. Although direct surgery is generally reported to have a better outcome, there is no consensus on the option which to use has been obtained so far. Endovascular treatment has the potential for incomplete occlusion and recanalization and the risk for ischemic complication. Owing to fine and tortuous feeding arteries and arteries such as ASA arising from the VA, which may present an acute angle, the endovascular access is considered difficult [2].

On the other hand, in the acute phase of hemorrhage, due to brain swelling, it is considered difficult to ensure the operative field. In addition, Song et al. [10] reported that, in most cases of AVFs at CCJ fed by ASA, the fistula was often situated on the ventral side of spinal cord, and it was almost impossible to expose and clip the aneurysm associated with the AVF during direct surgery, so endovascular embolization was a useful treatment.

Recent advances in endovascular devices have made it possible to improve the catheterization into small vessels and understand angioarchitecture more deeply by selective angiography. In particular, CCJ AVFs have complex vascular anatomy, thus selective angiography is essential for making a diagnosis of them [1]. In the present case, the micro catheter could be placed near the shunt, and the selective angiography made it possible to expect the safety range where NBCA did not migrate into the ASA. Additionally, based on the fusion image of left and right VA angiography, we could accurately grasp the shunt points and perform the successful treatment.

The presence of the aneurysm/varix has been considered the cause of SAH [1,^11^,12]. However, there is controversy about the need for occlusion of the aneurysm/varix. The development of the aneurysm/varix is consequence of hemodynamic and flow-related phenomena. Therefore, only shunt obliteration may be the effective treatment. In fact, it was reported that 5 patients showed spontaneous disappearance of the aneurysm/varix after only the shunt obliteration [13], [14], [15], [16]. In our case, we performed the glue embolization of the fistula up to the drainer vein, and postoperative angiography showed the disappearance of not only the fistula but also the varix.

In the previous review, transarterial embolization of CCJ AVFs with coil and NBCA were performed and favorable outcomes were obtained [13,17]. Lately, a nonadhesive embolic agent has been increasingly used for treatment of CCJ AVFs [18]. Because of the complex angioarchitecture of the CCJ AVF and the narrow safety zone for embolization, we believe that NBCA, an adhesive embolization agent, is more reliable than nonadhesive agents if the catheter can be guided close enough to the shunt. It should also be noted that NBCA induces inflammatory thrombosis, which can lead to complete occlusion even if partial embolization is achieved, but conversely, there is a risk of ischemic complications due to the development of thrombosis [19,20]. We consider that, when performing embolization, using the high-resolution setting of image acquisition, navigating the micro catheter to a position close to the site of the fistula, and using the balloon catheter as necessary to control NBCA are useful to avoid the embolic complications.

Despite selective angiography, it is difficult to comprehend all of small anastomoses. Therefore, it is possible to happen unexpected migration of embolic agents, which cause neurological deficit due to ischemic complications. Although it requires careful and sufficient consideration to select endovascular treatment for CCJ AVFs, it is useful in the acute phase of hemorrhage or depending on the location of the fistula. Like the present case, due to understanding of the detailed vascular anatomy around the fistula by selective angiography, endovascular treatment for CCJ AVFs may be performed more safely.

## Conclusion

Endovascular treatment for CCJ AVFs is expected from the point of view of minimal invasiveness and considered when it is difficult to approach the site of the fistula by direct surgery, and detailed understanding of the angioarchitecture is required. Due to further understanding of vascular anatomy and advances in endovascular devices, endovascular treatment for CCJ AVFs will become safer and more reliable.

## Patient consent

Complete written informed consent was obtained from the patient for the publication of this study and accompanying images.
